# Supplementary material for: A systematic scoping review and thematic analysis: How can livestock and poultry movement networks inform disease surveillance and control at the global scale?
Source: PLoS One. 2025 Jul 21;20(7):e0328518. doi: 10.1371/journal.pone.0328518 (PMC12279109; doi:10.1371/journal.pone.0328518)
Supplement: Attachment — Submitted filename: reviewSystematic.pdf [file pone.0328518.s003.pdf]

The article is very interesting and well-written. The authors conducted rigorous, extensive, and interesting work that needs to be shared. There are only a few minor points that I wish the authors would address.

In the abstract:

The sentence "The increasing threat of emerging infectious diseases affecting animal and human populations has prompted a critical examination of their origins and contact patterns driving disease transmission" could be misleading. " could be misleading. I usually associate transmission with a local event (a susceptible individual being infected by another) and use the term "propagation" when discussing epidemics that appear in different subpopulations. Similarly, contact patterns make me think of individual habits. If you could rephrase to focus more on the interaction between multiple locations, it would greatly improve readability.

The same goes for the sentence, "Five themes emerged: network structure (73.4%), epidemic modeling (35.0%), targeted control (31.5%), outbreak analysis (14.8%), and network inference." could be misleading. I suppose there is a certain degree of overlap among the articles. Perhaps it would be better not to show percentages.

In Section 3.2.1,

It would be nice to stratify Figure 3A by continent or economic area to show whether works in these areas are becoming more frequent.

Similarly, it would be nice to present a table similar to Table 3, organized by geographical continent or economic area.
