# Supplementary material for: A systematic scoping review and thematic analysis: How can livestock and poultry movement networks inform disease surveillance and control at the global scale?
Source: PLoS One. 2025 Jul 21;20(7):e0328518. doi: 10.1371/journal.pone.0328518 (PMC12279109; doi:10.1371/journal.pone.0328518)
Supplement: Attachment — Submitted filename: Response to Reviewers.docx [file pone.0328518.s004.docx]

## Response to Reviewer Comments

Manuscript Title: A systematic scoping review and thematic analysis: How can livestock and poultry movement networks inform disease surveillance and control at the global scale?

Manuscript ID: PONE-D-25-08168

Note: Line numbers provided to support our answers correspond with the highlighted areas in **Revised Manuscript with Track Changes*.docx*.**

### Editor

*“1. When submitting your revision, we need you to address these additional requirements (…)”*

**Author:** Formatting was updated as required.

*“2. Thank you for stating the following financial disclosure: [There is no specific funding for the scoping review hereby presented but the main authors' research is supported by the Agriculture and Food Research Initiative Competitive Grant no. 2022-68015-36628, awarded by the United States Department of Agriculture.]”*

**Author:** We have included the suggested statement “The funders had no role in study design, data collection and analysis, decision to publish, or preparation of the manuscript.” in the cover letter, as requested.

*“3.* *Please amend either the title on the online submission form (via Edit Submission) or the title in the manuscript so that they are identical. “*

**Author:** Title was amended to be consistent across document and online submission.

*“4. We note that Figure 3 in your submission contain [map/satellite] images which may be copyrighted. All PLOS content is published under the Creative Commons Attribution License (CC BY 4.0), which means that the manuscript, images, and Supporting Information files will be freely available online, and any third party is permitted to access, download, copy, distribute, and use these materials in any way, even commercially, with proper attribution. For these reasons, we cannot publish previously copyrighted maps or satellite images created using proprietary data, such as Google software (Google Maps, Street View, and Earth).”*

**Author:** Figure 3 is original, created by the main author using R software, therefore compliant with the CC BY 4.0. We have included more details in the “materials and methods” regarding its design [lines 193-194] and in the respective caption for Figure 3 [line 270].

[lines 191-192] - “Maps were created using sf (33), and maps (34) packages in R.”

[line 268 – Figure 3] - “Maps were created using sf (33), and maps (34) packages in R..”

### Reviewer #1

*“ This article is very interesting as it is providing a nice overview of the application of network analysis in veterinary epidemiology, the strengths and limitations that have been identified in this area. However, regarding the claimed benefits of identifying key nodes to guide risk-based surveillance for instance, or to tailor control measures, my question is: have these been demonstrated to date? It seems that the implementation of the outcomes of these studies with associated positive results has yet to be demonstrated. Perhaps this is due to the limitations that have been identified by the studies that are reported in the present paper. But it would add to the manuscript to have a brief discussion about this. Theoretically, it makes sense, but I have yet to see a publication showing its real-world application.”*

**Author:** We appreciate your comment; this is a great point. While multiple studies have demonstrated the “potential” of network analysis to inform disease control (e.g., identifying high-risk nodes and design/compare theoretically optimal interventions), we agree that evidence of real-world implementation remains limited. Most published examples rely on pre-defined modeling frameworks to simulate disease transmission.

We acknowledge that such simulations are based on assumptions that inherently limit their real-world generalizability (models are as good as the data we have). However, we also note that testing these interventions during ongoing outbreaks can be challenging due to the urgent nature of response efforts, and because interventions often occur at the regional level rather than allowing for controlled comparisons. Network studies like the ones investigated highlight the importance of detailed and high-quality movement data, which, when available, can be used to parametrize models more accurately to reflect true contact structures.

To address this point, we have highlighted the gap between theory and practice in the discussion [lines 548-558] and have softened the language throughout the manuscript (e.g. lines 505-508) to reflect that network-informed strategies are best viewed as complementary tools, whose effectiveness depends on data quality and operational context.

*“- Although there is mention that FMD 2001 likely led to a number of network analysis studies, I believe there are other factors that have contributed to the observed body of literature in this area. It would have been nice to show on Figure 4, or in the discussion, associated major disease events or changes in control policies, or data systems becoming online… that could explain the rise in network analysis studies:*

*o ASF in Eastern Europe as of 2014 and ASF in China in August 2018 – these events likely explain the observed rise in studies pertaining to swine;*

*o Cattle passport system with electronic database in the EU since 2000;*

*o Of course FMD in 2001 but also bluetongue perhaps in Europe since 2006-2010?*

*o HPAI in poultry; are peaks in studies associated with peaks in outbreaks globally?*

*o Ongoing tuberculosis control in various countries?*

*o Spread of PPR in Europe since around 2016?.”*

**Author:** We fully agree that several outbreaks and policy milestones—such as ASF emergence in Eastern Europe and China, the establishment of cattle passport systems in the EU, and the spread of HPAI etc. —have likely influenced the rise in network analysis studies across species. However, the authors had difficulties in selecting which events to choose and present if we are more comprehensive, and this ended up taking us outside of the scope of the manuscript, which is already quite long as is. For the sake of readability and focus, we chose to present the overall trends without linking them to specific outbreak timelines. However, we appreciate the reviewer’s point and believe future work exploring these temporal associations in detail would be interesting.

**Specific/detailed comments:**

*“Line 66 : Reference 8 is not a good example of “robust livestock and poultry traceability systems worldwide” but is a reference that mentions good examples of such systems. Suggest you list references that are direct examples of such robust systems.”*

**Author:** We have revised the sentence to include a more concrete example (Cattle Tracing System in the UK) and updated Reference 8 to a more appropriate source, as below.

[line 66]: “traceability systems worldwide, **such as the Cattle Tracing System in Great Britain (8).**”

*British Cattle Movement Service. CTSonline the easy way to check and report your cattle information. [cited 2025 Apr 21]. Cattle Tracing System (CTS). Available from: https://secure.services.defra.gov.uk/wps/portal/ctso*

*“Line 80: “… not only used to trigger hot spots…” . I am not sure what this means. The way that a network is organized as a result of the specific production system, may lead to the emergence of hot spots or super-spreaders that may be involved in pathogen transmission and their identification in a specific network may inform surveillance and control for targeted, risk-based, surveillance. I believe this is what you are trying to say? As I don’t know how the dynamics of the network could trigger hot spots…”*

**Author:** Thank you for the note. We agree that the original wording was unclear and have revised the sentence to replace “trigger” by “identify”.

[lines 80-83]: “These important dynamic characteristics could be not only used to identify “hot spots” or “super-spreaders” (i.e., key players) for pathogen transmission within these networks but also serve as an excellent source to inform disease surveillance and control in addition to more conventional methods.”

*“Line 85: animal transportation networks. There are animal movement networks that are not based on transportation. Transhumance, pastoral-type of systems where animals are moved on foot, sometimes across borders etc… Were these included in the scope, and if they were, perhaps there were none published? In the discussion, I think it is important to note if the majority of papers come from countries with similar types of production systems that are more apt to have movement databases than others. Regardless of the means to develop such systems, the type of production just doesn’t allow their implementation, i.e., small ruminants, which can explain why there are less papers published. Such analysis in thinking about why you have the descriptive statistics that you have would have brought an interesting discussion to the paper.”*

**Author:** Indeed, we did not exclude transhumance or grazing movements in our search criteria. However, our search strategy – as specified in the OSF-registered protocol – was structured around terms commonly associated with traceable or recorded movement data. We are aware that in some countries, grazing permits or similar forms of movement are integrated into traceability systems, but unfortunately, these are rarely available in reported data. As a result, it was not feasible to assess the relative contribution of these movement types across studies.

We agree that comparing production systems would enrich the interpretation of trends. To partially address this, our results discuss the methodology used by different studies to collect data as a proxy for underlying differences in traceability infrastructure—e.g., snowball sampling and surveys, which are typically used when data is lacking and was more commonly observed in low- and middle-income countries [lines 309-321]. While we prioritized reporting study-level strengths and limitations by country context, we note that more granular comparisons of production systems would be a valuable area for future research [lines 519-521].

*“Line 86-87: sentence doesn’t read well. Suggest: “One available piece of work focused on a review of the different network-based modelling frameworks used to represent animal movements (24).”*

**Author:** The sentence was revised to match the suggestion [lines 87-89].

*“Line 129: animal movement or transportation data. Again, please explain so the reader can understand what you mean, i.e., how they differ.”*

**Author:** We have revised the text to clarify that “animal movement data” in this review refers broadly to all types of animal relocations between premises, including those by truck as well as other forms such as grazing movements.

[lines 131-134] – “The research papers had to use or provide insights into the applications of network analysis on studying disease dynamics, by applying these techniques to animal movement data, whether involving transportation by vehicle, grazing, or other forms of relocation of livestock and poultry between facilities.”

*“Line 199: animal movement studies is used here. Please be consistent.”*

**Author:** The wording has been revised as below.

[line 449-50] - “This review identified both strengths and limitations in the application of network analysis to livestock and poultry movements.”

*“Line 251: could have the reference here since mentioning a specific article?”*

**Author:** Thank you for pointing this out! We agree that is valuable to add the reference for this case and have included it in the text [line 265 – reference 40].

*“Table 1: would help the reader if added lines separating the countries as it was difficult to follow.”*

**Author:** Table was corrected [line 287].

*“Line 322: It would be of added value to know if the papers used directed or undirected networks in their studies. The use of the term “edge” in the manuscript can create confusion. The more precise term to use in directed networks is “arc” to represent the directional movement from one node to another. I suspect that the vast majority of the studies that you considered are based on directed network, but this should be mentioned in the descriptive statistics.*

*- This raises one question. A number of papers that have been published explored different network analysis measures to determine which could be the more useful to meet certain objectives: predict epidemic size, construct network structures on which to model disease spread etc.. It seems this theme was not picked up in your study where you focus on network structure, not network measures. There has been a body of work looking at applying social network analysis measures and relating them to veterinary epidemiology and it seems this is not reflected in your study. Perhaps this needs to be discussed?”*

**Author:** We have now included this missing piece in the results as follows:

[lines 340-342] – “Notably, almost all studies (n= 202, 99.5%) represented animal movements as directed networks, reflecting the origin and destination of each movement; only one study used an undirected approach (41).”

Regarding terminology, we opted to retain the term “edge” throughout the manuscript for consistency with the broader network analysis literature and to align with the terminology used in our reference book “*Networks*” by Mark Newman. We hope this choice maintains accessibility for readers while still conveying the intended meaning.

*Newman, Mark, Networks, 2nd edn (Oxford, 2018; online edn, Oxford Academic, 18 Oct. 2018), https://doi.org/10.1093/oso/9780198805090.001.0001.*

*“Line 380: replace “animal networks” with “animal movement networks”*

**Author:** Wording was replaced as suggested [line 402].

*- Data collection and quality: in the discussion, there should be mention of small ruminants where sure, it can be a matter of veterinary resources and infrastructure that complicate the ability to obtain information on this population, but also, it is the structure of that sector that also makes it very difficult to track? Yet, they are extremely important components of the global spread of diseases, think FMD, PPR etc… This should be covered in the discussion, there are certainly technological challenges at gaining traceability for this population.”*

**Author:** Thank you for this important point. We agree that small ruminants are underrepresented in network analysis studies, despite their significant role in the global epidemiology of transboundary diseases such as FMD and PPR. This underrepresentation could reflect both limited traceability infrastructure in many regions and the inherent challenges of tracking animals managed in more extensive or informal production systems. In fact, our results support these assumptions. We have now added a sentence [lines 539-542] in the Discussion to reflect these considerations, and to highlight the need for alternative data collection strategies tailored to these sectors.

[lines 539-542] – “Similarly, the underrepresentation of small ruminant’ networks may reflect not only data limitations but also the inherent challenges of tracking animals in more extensive, pastoral or smallholder settings, where centralized data collection is often not feasible.”

*“Line 467: in this discussion it must be added that animal movements, while important, are one pathway for disease transmission and that others such as fomites, wildlife, etc… are important as well. By focusing only on animal movement, we do not have full comprehension of the network structure.”*

**Author:** We agree that this was pointed out in the results and have now included a note in the discussion as follows:

[lines 547-549] – “Moreover, it is also important to consider other relevant transmission routes, such as fomites, wildlife, or environmental reservoirs, that may contribute to pathogen dissemination.”

*“Line 467-479: I don’t see the point of this example which is not discussed in the rest of the manuscript. Reference 75 can be used to represent public private and academic partnerships.”*

**Author:** We have now removed that sentence and incorporated the reference within the previous sentence [line 500].

*“Line 480-484: these benefits keep being mentioned and are theoretical at this time. Are there any papers that actually have shown how implementing the findings from animal movement studies have benefited either early detection or control of diseases?”*

**Author:** We have revised the paragraph in question to soften the language and better reflect the largely theoretical or model-based nature of the findings. Later on, we clarify that most examples rely on simulation-based studies and note the logistical and policy-related challenges that limit translation into practice, as follows:

[line 551-561]: “Finally, while many studies proposed network-informed interventions, we must note that few have demonstrated their implementation in real-world scenarios. Most examples in the literature involved scenarios based on network-informed transmission models which, while valuable, are inherently limited by the assumptions they rely upon and the quality of input data. The challenge of translating these insights into practices reflects both logistical difficulties in outbreak settings and the complexity of coordinating policy shifts based on predictive tools. Nonetheless, the ability to incorporate detailed movement data into disease modeling remains a promising tool.”

*“Line 524 – 527: I think that the first part of this sentence is true (identifying potentially high-risk components – though components in network analysis has a meaning, perhaps find another word), theoretically, but the second part of the sentence has not been demonstrated in animal movement network analysis studies (underscoring the importance of targeted surveillance and biosecurity measures, as opposed to other approaches).”*

**Author:** Thank you for this comment. We agree that the word “components” is misused here and have replaced it. We have also rephrased the sentence to ensure that we emphasize “network analysis potential” in theory:

[lines 583-586] – “In conclusion, network analysis has proven a powerful tool in identifying at-risk nodes or groups within livestock and poultry movement networks, offering promising theoretical insights for targeted surveillance and biosecurity strategies that may outperform traditional approaches.”

*“The limitations that researchers identify, especially in terms of completeness of data to really completely understand and identify high-risk nodes mean that there has not been yet implementation of policies, surveillance and control measures solely relying on animal movement network findings. Identification is not the same as modelling disease spread as well.*

*Biosecurity helps all nodes, not just highly connected ones and I would doubt that any veterinary services around the world would target biosecurity measures only at high-risk nodes derived from incomplete networks. Until complete networks are available, I believe all this work remains informative and theoretical, without implementation. As a result, the language should be softened in this paper.”*

**Author:** We appreciate your thoughtful review. We carefully revised the language throughout the manuscript, particularly discussion and conclusion, to add a more cautious tone. Specifically, we emphasized that network analysis offers promising insights and made sure we acknowledged that its real-world implementation remains theoretical.

### Reviewer #2

*“1. Although the authors appropriately acknowledged the limitations of network analysis in their conclusions, I encourage them to include a few sentences discussing the limitations of the scoping review itself in identifying all relevant prior applications of network analysis. For example, I was unable to find references to two earlier technical reports on animal movement network analysis conducted for the European Union and Denmark. It appears that the authors may have been constrained by the availability of sources in the databases they accessed.”*

**Author:** We appreciate the thoughtful insights and thank you for taking the time to review our manuscript. While we recognize that there is always a possibility of missing relevant literature—particularly grey literature or non-indexed reports—we designed our search strategy to be as inclusive as possible and followed a predefined protocol. Given the study period and the scope of our search across multiple databases, we are confident that most peer-reviewed studies meeting our criteria were captured. Nonetheless, we have added a sentence in the limitations section acknowledging this potential constraint, as follows:

[567-569] – “While our search strategy was designed to be as inclusive as possible, we acknowledge that there is always a possibility of missing some studies, especially grey literature and non-indexed reports. (…)”

*“2. Based on their findings, the authors might consider adding a few sentences offering recommendations on when and how to apply network analysis, including any requirements or considerations. Such guidance would be valuable for future users and decision-makers, especially if supported by evidence from prior work.*

*I wish the authors continued success in maintaining this high standard of research to support better disease management in both human and animal populations.”*

**Author:** We have now added a brief statement in the discussion [514-521] highlighting key considerations for applying network analysis, including the importance of data availability, production system characteristics, and surveillance goals. We appreciate your success wishes and likewise wish you all the best in your continued great work!

[514-521] – “Based on the findings, we recommend future research to clearly define nodes in alignment with the relevant epidemiological units. While premises offer more granular resolution, regional-level nodes are often the only available choice, and may suffice when disease mitigation strategies are implemented at broader administrative levels. The selection of network metrics should also be guided by the specific research question, as different metrics offer distinct information. These choices should be tailored to the production system, pathogen characteristics, and the temporal and structural properties of the network of interest.”
