# Supplementary material for: A systematic scoping review and thematic analysis: How can livestock and poultry movement networks inform disease surveillance and control at the global scale?
Source: PLoS One. 2025 Jul 21;20(7):e0328518. doi: 10.1371/journal.pone.0328518 (PMC12279109; doi:10.1371/journal.pone.0328518)
Supplement: Attachment — Submitted filename: Response to Reviewers R2.docx [file pone.0328518.s005.docx]

## Response to Reviewer Comments

Manuscript Title: A systematic scoping review and thematic analysis: How can livestock and poultry movement networks inform disease surveillance and control at the global scale?

Manuscript ID: PONE-D-25-08168

Note: Line numbers provided to support our answers correspond with the highlighted areas in **Revised Manuscript with Tracked Changes*.docx*.**

### REVIEWER #1

*“Dear Author,*

*Thank you for your review of the comments I put forward. I am in agreement with your answers and your modifications in the manuscript that has improved as a result. Just a few minor things:*

*Line 86 and 94, check again the use of transportation instead of movement.”*

**Author:** Thank you for the encouragement and we appreciate again your insightful comments! I have corrected lines 87 and 95 to read “movement” networks instead of transportation.

*“Line 203 , perhaps it is better to say application of network analysis to (instead of in) animal movement data?”*

**Author:** We agree, thank you! I have corrected the wording [line 206].

*“Line 515:* *are recognized as potential (word added) hotspots? In your references, perhaps two demonstrate the actual risk whereas others suggest based on network?“*

**Author:** You are correct; some references solely suggest this association based on theoretical risk. We have revised the sentence for improved clarity and to better reflect the existing literature:

[lines 512-515] – “Markets, in particular, have been linked to disease transmission risks - some studies demonstrating direct associations with outbreaks (64,83,84), while others highlight their structural role and centrality within movement networks as indicative of potential risk (81,82) - yet data from these settings remain scarce.”

### Reviewer #2

*“ Congratulations for good submission.”*

**Author:** We thank you for the words of encouragement!

### Reviewer #3

*“The article is very interesting and well-written. The authors conducted rigorous, extensive, and interesting work that needs to be shared. There are only a few minor points that I wish the authors would address..”*

**Author:** Thank you for taking the time to review our manuscript. We appreciate your support and will address the comments below.

*“In the abstract:*

*The sentence "The increasing threat of emerging infectious diseases affecting animal and human populations has prompted a critical examination of their origins and contact patterns driving disease transmission" could be misleading. " could be misleading. I usually associate transmission with a local event (a susceptible individual being infected by another) and use the term "propagation" when discussing epidemics that appear in different subpopulations. Similarly, contact patterns make me think of individual habits. If you could rephrase to focus more on the interaction between multiple locations, it would greatly improve readability.”*

**Author:** We agree that the original sentence could be misinterpreted and have reviewed it accordingly:

[lines 20-21] - “The increasing threat of emerging infectious diseases affecting animal and human populations has prompted closer investigation into how movement-linked interactions contribute to geographic spread of pathogens.”

*“The same goes for the sentence, "Five themes emerged: network structure (73.4%), epidemic modeling (35.0%), targeted control (31.5%), outbreak analysis (14.8%), and network inference." could be misleading. I suppose there is a certain degree of overlap among the articles. Perhaps it would be better not to show percentages.”*

**Author:** Thank you for the comment, we have revised the sentence and removed the percentages while maintaining the thematic structure [lines 36-37].

*“In Section 3.2.1,*

*It would be nice to stratify Figure 3A by continent or economic area to show whether works in these areas are becoming more frequent. Similarly, it would be nice to present a table similar to Table 3, organized by geographical continent or economic area.”*

**Author:** Thank you for the suggestion. While stratifying results by continent or economic area was not a predefined aim of our study, we recognize the importance of highlighting global disparities in research output. Our analysis revealed that most studies originated from high-income countries, underscoring a notable geographic and economic imbalance in the application of movement network approaches (see Table below summarizing the number of studies based on the World Bank’s FY 2023 income thresholds - most recent to date).

| **Income Group (GNI)** | **No. of studies** | **Continent** | **Count** |
| --- | --- | --- | --- |
| High income (>$14,005) | 133 | Europe | 83 |
|  |  | Americas | 41 |
|  |  | Oceania | 6 |
|  |  | Asia | 3 |
| Upper-middle income ($4,516 and $14,005) | 48 | Asia | 25 |
|  |  | Americas | 18 |
|  |  | Africa | 3 |
|  |  | Europe | 1 |
|  |  | Oceania | 1 |
| Lower-middle income ($1,146 and $4,515) | 22 | Africa | 10 |
|  |  | Asia | 9 |
|  |  | Oceania | 3 |
| Low income (<$1,145) | 10 | Africa | 10 |
| Unclassified | 1 | Africa | 1 |

Note: unclassified did not have available GNI (gross national income) per capita.

Although we opted not to include additional maps or figures, we have incorporated a brief statement in the manuscript to reflect this key observation. We hope this provides helpful context on the global distribution of network-based research.

[lines 277-279] – “The vast majority of studies (n=181, 89.2%) were conducted in high-income (n=133) or upper-middle-income countries (n=48), with Europe and the Americas being the most represented continents (n=124, 61.1%).”
